# Exploring Inhibitory Control Processes in Highly Superior Autobiographical Memory (HSAM): A Single Case Study

**DOI:** 10.5334/joc.421

**Published:** 2025-01-21

**Authors:** Jessica Talbot, Daniele Gatti, Marta Boccalari, Michela Marchetti, Danilo Mitaritonna, Gianmarco Convertino, Mara Stockner, Giuliana Mazzoni

**Affiliations:** 1 Faculty of Medicine and Psychology, University of Sapienza, Rome, Italy; 2 Department of Brain and Behavioral Sciences, University of Pavia, Pavia, Italy

**Keywords:** Highly superior autobiographical memory (HSAM), autobiographical memory, cognitive control, inhibition, executive functioning

## Abstract

Individuals who possess a Highly Superior Autobiographical Memory (HSAM) can remember their own lives in exceptional detail, retrieving specific autobiographical events in response to dates (e.g., 15^th^ April 1995). The phenomenon remains extremely rare, and little is known about why these individuals can remember substantially more than the general population, without being continually flooded by past memories. According to the *cognitive inhibition dependency hypothesis*, inhibitory processes modulate general autobiographical memory by determining which memories will (and will not) enter one’s consciousness. We hypothesised that these control processes are amplified in HSAM, protecting them from being overwhelmed by their abundance of memories. To explore if cognitive inhibition is exceptional in HSAM, a single case with HSAM (DT) and 20 matched controls completed a battery of 6 tasks assessing various aspects of inhibition (e.g., memory, prepotent motor responses). Participants also completed a screening for obsessive compulsive disorder and autism. Results indicate that DT’s inhibitory functioning is comparable to that of the typical population, and thus not exceptional. We conclude that inhibition is unlikely to be the best explanation for extraordinary remembering and add to the growing body of literature that HSAM can occur in the absence of clinical symptomatology. Results are discussed in relation to future directions of HSAM research.

## Introduction

Forgetting is broadly accepted by researchers to be a fundamental (and adaptive) component of *ordinary* autobiographical memory functioning (Maxcey et al., 2019; Nørby, 2015; Schacter, 2022; Somos et al., 2022). So-called active forgetting is deemed a deliberate neurocognitive process that involves the prioritised encoding and retrieval of selected material (Anderson & Hulbert, 2021; Anderson & Hanslmayr, 2014). In fact, losing some information is believed to serve countless advantages, such as providing serenity, stability and clarity (i.e., the Cardinal Virtues of Forgetting, see Fawcett & Hulbert, 2020).

Failures to retrieve information may be common to ‘normal’ memory, but in the last 20 years a form of exceptional memory called Highly Superior Autobiographical Memory (HSAM) (Frithsen et al., 2018) has been identified which challenges the upper limits of “normal” autobiographical remembering. HSAM, or as it was originally coined, hyperthymesia (Parker et al., 2006), is a label given to a very small group of individuals worldwide (Patihis, 2015) who are unable to forget. HSAM individuals demonstrate a specific aptitude for recalling personal events in response to date cues (Ally et al., 2013; De Marco et al., 2021; Gibson et al., 2022; Talbot et al., 2024). Importantly, HSAM is not a form of savant syndrome (e.g., De Marco et al., 2015), where individuals possess a neurodevelopmental condition like autism spectrum disorder (Hughes et al., 2018), which coincides with a truly extraordinary ability for one or more domains, such as memory (Neumann et al., 2010). The phenomenon is also distinct from other groups of exceptional memory, such as Memory Athletes (Dresler et al., 2017), that implement methodical mnemonic strategies to achieve their remarkable memory feats.

According to one theory of autobiographical memory functioning, entitled Transition Theory (Brown, 2016; Brown 2021), much of a ‘normal’ person’s life is repetitive and mundane (e.g., commuting) (Brown, 2023). Thus, many memory traces are quickly forgotten, unless they are particularly distinct and/or “worthy” of implicit or explicit rehearsal (Brown, 2023). In HSAM, however, participants remember regardless of the monotony of such events. HSAM individuals can provide highly detailed (LePort et al., 2016) recollections for most days of their life (Parker et al., 2006; Talbot et al., 2022) and can also access these memories exceptionally quickly in response to a given cue (Mazzoni et al., 2019). Their accuracy at providing verifiable information about the dates (e.g., something that happened in the news that day, Parker et al., 2006) is extremely high, indicating a unique capacity to efficiently search their own memory and retrieve a relevant memory.

One potential explanation for how HSAM individuals can quickly retrieve the “correct” memory for a date cue, whilst retaining *substantially* more memories than other people, is that HSAM is characterised by enhanced functioning of an aspect of cognitive control (i.e., cognitive inhibition). Cognitive inhibition refers to the deliberate suppression of material, including thoughts, memories, and impulses, that are deemed irrelevant to the present goal (Hasher & Zacks, 1988; See Gohier et al., 2009 for clinical evidence). Tuning out stimuli that are not adaptative for the task being undertaken, improves a persons’ capacity to adequately complete required cognitive processes (Robledo-Castro et al., 2023). As HSAM individuals have more stimuli to tune out, and can access memories much faster, we hypothesised that their inhibition may be enhanced.

HSAM individuals can also live their life without being continually flooded by the countless past events they remember entering their mind (but cf. Parker et al., 2006 for an example of a HSAM individual who is consumed by their memory). What prevents the vast number of past memories inundating the mind of individuals with HSAM remains largely unknown. Cognitive inhibition has been considered in relation to the mechanisms contributing to involuntary autobiographical memory retrieval (Barzykowski et al., 2019). Involuntary autobiographical memories are past events that enter one’s mind spontaneously, without a deliberate retrieval attempt, in response to internal or external cues (Barzykowski & Mazzoni, 2021; Berntsen, 2010; Laughland & Kvavilashvili, 2024; Mace, 2007; Vannucci et al., 2014). As Berntsen (2009, pp. 98–99) describes, listening to a thunderstorm outside can unintentionally trigger a past memory which also involves a thunderstorm. If something as simple as the weather can initiate retrieval of an involuntary autobiographical memory, researchers have debated why individuals are able to carry out various daily activities without being overwhelmed by such thoughts (Vannucci et al., 2015).

According to the *cognitive inhibition dependency hypothesis* (Barzykowski et al., 2019), cognitive inhibitory processes may modulate involuntary mental time travel (i.e., both past retrieval and future thinking, e.g., Finnbogadóttir & Bernsten, 2013). The most influential theory of autobiographical memory, the self-memory system (Conway & Pleydell-Pearce, 2000), theorises that various mental representations are continually being activated at the lowest hierarchal level. Only some of these activated memories reach consciousness, whilst others are suppressed by executive control processes, and thus, frequency of involuntary retrieval could therefore be dependent on effective or ineffective functioning of inhibitory control mechanisms (Barzykowski et al., 2019). Similarly, Hasher et al. (2007) state that inhibition functions, in part, to suppress irrelevant interference, meaning inhibitory deficits (or depleting inhibitory control resources) could lead to greater involuntary autobiographical memories.

Importantly, the work of Barzykowski et al. (2019) found that inhibitory control did not affect the frequency of involuntary memories in typical adults, however, as HSAM is atypical, we postulated inhibition could extend to the topic of *exceptional* autobiographical memory to support enhanced encoding and retrieval. As HSAM individuals retain significantly more personal events than controls; we assumed they a). have a greater number of memories which “match” internal and external cues, and b). that they have a greater ability to control them. Testing this theory could be particularly useful as the precise cognitive mechanisms supporting HSAM functioning is poorly understood, with several studies indicating that HSAM individuals perform normally in various non-autobiographical memory tasks (Daviddi et al., 2022; LePort et al., 2012; LePort et al., 2017). As HSAM individuals can live their lives unburdened by their ability (e.g., DT is a successful journalist, Talbot et al., 2022), we hypothesised HSAM individuals may possess more efficient control processes which supress unwanted memories from continually flooding their present.

To test the prediction that enhanced inhibitory processing is a feature of HSAM, we administered a battery of six cognitive inhibition tasks to compare HSAM performance to matched controls. To paint a thorough (and as broad as possible) picture of overall performance, the battery was designed to assess various aspects of inhibition (e.g., memory, prepotent motor responses). Alongside an exceptional memory capacity, some HSAM participants have been found who excel at their personal memory but concomitantly exhibit symptoms indicative of clinical conditions, such as obsessive-compulsive disorder (OCD) (Ford et al., 2022; Gibson et al., 2022; Parker et al., 2006; LePort et al., 2017; Santangelo et al., 2018), or autism spectrum disorder (Ford et al., 2022; Gibson et al., 2022). Such symptomatology could contribute to a remarkable ability to remember, via a compulsive tendency to remember the past in OCD (Palombo et al., 2018) or due to savant syndrome (Hughes et al., 2018). These clinical profiles are not seen in all HSAM participants and recent, albeit rare, evidence now supports that HSAM can occur in the absence of any clinical abnormalities (De Marco et al., 2021; Mazzoni et al., 2019; Santangelo et al., 2021). This development of knowledge appears to indicate there may be distinct subtypes of HSAM that vary in clinical profiles and could be sustained by different underlying mechanisms. As these conditions have been found to be linked to impairments in cognitive inhibition (e.g., Enright & Beech (1993) for OCD evidence, Agam et al. (2010) for evidence in autism), we opted to implement a single case design that involved a clinically healthy HSAM participant who has never received a diagnosis of either condition. In this way, we could initially explore non-clinical HSAM cognitive inhibitory processes, rather than disentangle possible clinical mechanisms. To provide evidence that the HSAM participant (DT) fell in the non-clinical range at time of testing (considering some symptoms, particularly for OCD, can be transient) all participants additionally completed an OCD and autism screen.

## Methodology

### Participants

DT is a physically, neurologically and psychologically healthy, 33-year-old male with HSAM that has been previously described in the literature (Talbot et al., 2022; Talbot, Gatti et al., 2024). He is Italian, right-handed, and is educated to master’s level.

To explore cognitive inhibition abilities in HSAM, DT’s performance was compared to 20 non-clinical, Italian-speaking control participants (See Crawford et al., 2006 and Crawford & Garthwaite, 2012 for a discussion regarding control sample size for single case research). Controls were matched to DT in relation to biological sex (male: n = 20), age (range = 30–34) and education level (all participants held a master’s degree/19 years of education). Control participants were recruited via a poster advertisement that was shared widely on social media. The poster requested individuals who i). matched the aforementioned age, sex and education background, ii). that were interested in completing a behavioural psychological task and iii). had computer access, to contact the third author to schedule testing.

During a preliminary subjective screen, control participants were asked if they believed they could be HSAM (i.e., they possessed an ability to remember most days of their life in response to dates). All control participants self-reported they were unable to retrieve date related memories in excess and believed their memory was “average”.

Participants signed informed consent to complete this research, which was approved by the Ethics Committee in the Department of Health, Dynamic and Clinical Psychology at Sapienza, University of Rome. No participants received monetary compensation for their involvement.

### HSAM screen

As previously described (Talbot et al., 2022), DT successfully completed a standardised 3-stage assessment for HSAM in January 2022. In Stage 1, the Hull Memory Screening Questionnaire (HMSQ) (Mazzoni et al., 2019) was administered via Microsoft Teams. His responses were consistent with answers of a HSAM participant and therefore he was invited to complete the more challenging: Stage 2 – Public Events Quiz (PEQ) and Stage 3 – the Random Dates Quiz (RDQ) (LePort et al., 2012).

The PEQ is a 30-item questionnaire that asks participants about important national and international public events. 15 questions provide a date (11^th^ of July 2021?) and ask participants to provide the title of the public event which happened on said date. A point is given if a correct answer is provided, and an additional point is given if the participant can also state the associated day of the week. 15 questions ask the participants to provide the date and day of the week of a public event (e.g., What date was the death of American citizen George Floyd?) (Talbot et al., 2022). For these questions, a point is awarded for each correct day, month, year, and day of the week. If participants score more than 50%, they are invited to complete the RDQ (DT’s score = 90.09%).

In the RDQ, a date is presented, and participants are required to provide a specific personal memory from that date. They receive one point for a specific event and a further point for providing the day of the week. A third point is awarded if participants can correctly provide a verifiable event from that date (e.g., something that happened in the news that can be verified on a news website). If participants score more than 65%, they are considered to have HSAM (DT’s score = 93.33%).

### Procedure

Participants completed a one-off Zoom call lasting approximately 60 minutes. Within the testing session, 6 tasks assessing various aspects of cognitive inhibition and 2 clinical questionnaires were administered by the third author in a fixed order (as presented below). Between each task participants were given a short break. Experimental tasks were programmed on PsychoPy (Peirce et al., 2019) and uploaded to Pavlovia for distribution, except the Part-Set Cuing task which was built via Google Forms. Prior to testing, participants were informed that the tasks would be cognitively demanding so they should be arranged for a date that they expected to be focused. All participants were tested in the afternoon (between 14:00 and 18:30).

### Arrow-Based Simon Task (Simon & Rudell, 1967)

Trials began with a blank black screen for 500 ms, followed by a white fixation cross in the centre of a black screen for 500 ms. Then a white arrow positioned in the left- or right-hand side of the screen appeared. Participants were instructed to indicate with the ‘A’ key if the arrow pointed towards the left of the screen or the “L” key if the arrow pointed towards the right of the screen, regardless of where the arrow was positioned. Arrows were displayed until a keyboard response was made or for a maximum of 5,000 ms in the case of no response. In total there were 200 trials. In congruent trials (50%), the position of the arrow on the screen matched the direction the arrow pointed (e.g., arrows pointing to the right placed on the right side of the screen, 25% of trials). In incongruent trials, the arrow position did not match the direction of the arrow (e.g., arrows pointing to the right placed to the left side of the screen, 25% of trials). Participants were instructed to keep their left index finger over the ‘A’ key and right index finger over the ‘L’ key for the duration of the task to ensure the most accurate response times could be recorded. Dependent variables were both accuracy and reaction times for congruent and incongruent trials.

### Part Set Cueing (Andrés & Howard, 2011)

To assess memory inhibition, the part set cueing task was administered. The task involves 3 phases, each involving a list of 24 semantically related words. Participants were informed they would hear a list of words and their task was to focus as much as possible on remembering each word, as they would be asked to retrieve them afterwards. Each word list was pre-recorded and was read out loud at a rate of 1.5 seconds per word, with the assistance of a metronome. The first list category was “animals”. After hearing the list, participants typed every word they could remember into a blank Google Form. For list 2 (fruits and vegetables), the Google Form they completed included 33% of the words from the original list and for List 3 (clothing) the Google Form included 66% of the words from the original list. Participants had a maximum of 4 minutes to type everything they could remember per list. For each phase, accuracy and number of intrusions was recorded.

### Go/No-Go Task (Donders, 1969)

Participants completed a 400-trial version of the Go/No-Go task. Each trial began with a 500 ms blank screen, followed by a red (75% of trials) or green (25% of trials) square that appeared in the centre of the screen for a fixed timing of 500 ms. Participants were instructed to do nothing when they saw a red square (No-Go trials) and to press the spacebar as quickly and accurately as possible when they saw a green square (Go trials). Participants were instructed to keep their index finger over the spacebar for the duration of the task to ensure the most accurate response times were recorded. Dependent variables were both accuracy and reaction times for Go trials.

Due to technical issues, Go/No-Go data of one control participant was not recorded, therefore analyses were run comparing DT to 19 controls.

### Hayling Sentence Completion Test (HSCT) (Burgess & Shallice, 1996)

Participants completed the Italian version of the HSCT (Scarpazza et al., 2023). 30 sentences were presented that each had the final word missing. Participants were instructed to state out loud a suitable missing word as quickly as possible. In Phase 1 (15 sentences), participants were instructed to provide a word that completed the sentence properly, in both a semantic and grammatical sense (e.g., Sentence: “People that cannot see well wear…”, Answer: “glasses”). In Phase 2 (15 sentences: inhibition phase) participants were instructed that a suitable word was a word that was illogical regarding meaning, but correct in a grammatical sense (e.g., Sentence: “Hens produce…”, Answer: “bananas”). All sentences were pre-recorded and played via Pavlovia to ensure consistency in administration. In both conditions, response times and accuracy were the dependent variables. 1 point was awarded for a completely correct answer, 0.5 points were awarded for a semi-correct answer (i.e., words semantically related to the proper phase, for example, “You must stop at the traffic light when it’s green”), and 0 points for an incorrect answer.

### Stroop Task (Stroop, 1935)

In Phase 1, a list of 30 words in black font was presented. Each word was a colour and participants were instructed to read the list sequentially. In phase 2, 30 rectangles of various colours were presented, and participants were instructed to state the colour of each rectangle sequentially. In Phase 3 (inhibition), a list of 30 written colours in various font colours was presented. Each written word was incongruent with the colour of the font. Participants were required to inhibit the written word and state the colours that were seen. For all phases, participants were instructed to speak as quickly as possible and continue to the next item if a mistake was made. Response times and accuracy were recorded for each phase.

### Open-Source Anticipated Response Inhibition Task (OSARI; He et al., 2022)

Participants’ ability to inhibit prepotent motor responses was measured using the OSARI. The task was built using the open-source Python library provided by He et al. (2022). In the centre of a black screen, a white vertical rectangle is presented. When the spacebar is held down the rectangle begins to fill in blue, at a fixed rate from the bottom. On Go trials (75% of trials), the goal is to release the spacebar when the blue line reaches the target markers (which are positioned at 800 ms). On Stop trials (25% of trials) the blue line stops before it reaches the target markers, and participants are required to inhibit the initiated response to release the space bar (i.e., they must continue to press the spacebar). Participants completed a practice and 3 testing blocks of 80 trials each. The Stop Signal Delay (SSD) was fixed at 500 ms. Dependent variables were Go and Stop accuracy, successful Go and failed Stop reaction times.

### Obsessive Compulsive Inventory – Revised (Foa et al., 2002)

All participants completed the 18 statement Obsessive Compulsive Inventory – Revised (OCI-R) Questionnaire to investigate the presence of obsessive-compulsive behaviour and thoughts. The scale is divided into 6 subscales (1. Cleanliness, 2. Obsession, 3. Order, 4. Checking, 5. Neutralisation, 6. Hoarding). Participants report on a scale from 0 (not at all) to 5 (extremely) how much the experience contained in the item has distressed or annoyed him/her in the last month. The scores in each item are added together. The OCD cut-off score was set at ≥ 21. In the present study, the Italian version of the OCI-R was used which was validated using both clinical and control populations (Sica et al., 2009). The tool has been found to possess good internal consistency and test-retest reliability. Specifically, the alpha value for the total scale was found to be high (0.85) and all Cronbach’s alpha coefficients for the six subscales exceeded 0.7, except for washing (Cronbach’s alpha = .60) and mental neutralizing (Cronbach’s alpha = .61), in a sample of 340 Italian participants. The Cronbach’s alpha in the present study sample was 0.83.

### Autism-Spectrum Quotient Test (Baron-Cohen et al., 2001)

All participants completed the Autism-Spectrum Quotient (AQ) to investigate autistic behaviours and thoughts. The scale consists of 50 sentences, to which the participant responds their degree of agreement on a scale from 0 (definitely disagree) to 3 (definitely agree). The cut-off score was set as ≥ 26. The AQ has been translated into multiple languages (for a review, see Ruzich et al. 2015) and in the present study the Italian version was used (Ruta et al., 2012). This version has been tested on a large sample (*n* > 500) of Italian participants and has been found to be a cross-culturally reliable tool, replicating the pattern of findings previously observed in a sample from the United Kingdom (Wheelwright et al., 2010). The Cronbach’s alpha in the present study sample was 0.81.

### Data preparation and analysis

Recorded verbal answers (i.e., HSCT, Stroop Task), written answers (i.e., Part Set Cueing), and selected questionnaire responses (i.e., OCI-R, AQ) were manually scored by the third author. Tasks that involved basic keyboard responses (i.e., Arrow-Based Simon Task, Go/No-Go, OSARI) included automatic response time recording and automated accuracy scoring. The HSCT and Stroop Task additionally included built-in microphones which automatically documented response times. In preparation for data analysis, mean ratings for each dependent variable (accuracy and response times) were calculated for each participant.

DT’s performance in each inhibition task was compared to controls by running modified t-tests that have been designed specifically for case-control comparisons (Crawford and Howell, 1998; Crawford et al., 2010; Crawford et al., 2011). Alternative methods, such as comparing a single case’s converted *z-*score to controls, have been criticised as they consider the control sample (which are usually *N*s < 20) as a population (not as *sample* statistics) (Crawford et al., 2006). In Crawford and colleagues (2006) method, the *t-*distribution (with *n* – 1 degrees of freedom) is used, instead of the normal distribution, to estimate the irregularity of a single case’s score, and examine whether it significantly differs to control sample scores (Crawford et al., 2006). The test uses the i). single case’s score, ii). the mean and standard deviation scores for the control sample and iii). the size of the control sample. This frequentist analysis was run using the *TD* function of the Singcar R package (Rittmo & McIntosh, 2021) on RStudio (R Studio Team, 2020). In addition to the *p* value, it also estimates the percentage of the control population exhibiting a higher or lower performance score (alongside confidence limits of this value), and provides point and interval estimates of effect sizes (Crawford et al., 2010).

Additionally, for each dependent variable included, we also estimated an intercept-only linear model (i.e., a null model) and a linear model having the group (HSAM vs. control) as categorical factor. Previous studies have indeed demonstrated that linear models and the Crawford and colleagues t-test are generally equivalent (Huber et al., 2015). From these two models we extracted the Bayesian Information Criterion (BIC), which is a measure of quality of the fit of a given model (Neath & Cavanaugh, 2012). BICs of two models fitted on the same set of data can be compared by computing the Δ between the two and then the Bayes Factor (BF) as the exponential of the ΔBIC halved (Wagenmakers, 2007). In the present analysis, BFs above 1 indicate evidence for the null hypothesis and BF below 1 indicate evidence for the alternative. Generally, BFs above 3 are considered indicative of moderate evidence in favor of the null hypothesis, but since BFs provide a continuous measure (and not dichotomic as in the case of p-values (e.g., Benjamin et al., 2018), even in the case of 1 < BF < 3, looking at the BF can provide informative results (Dienes, 2014).

## Results

**Figure 1:**
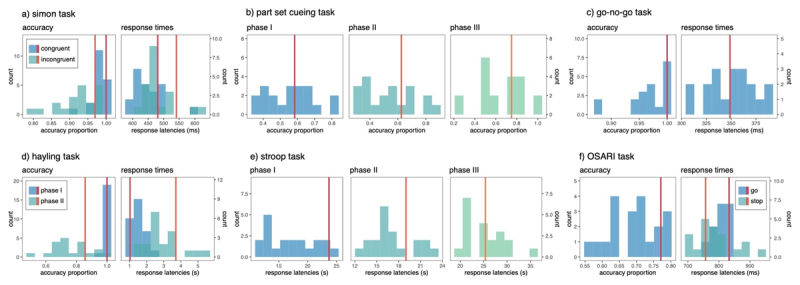
Overview of inhibition task results (accuracy and RT). DT’s performance relative to the control sample is depicted by red vertical lines. Stroop accuracy is not included as performance for all participants was at ceiling.

DT did not perform significantly better than controls in any inhibition task, in relation to accuracy (Table 1). He was also not significantly quicker (or slower) than controls at completing any of the inhibition tasks, regardless of trial congruency (all *p*s > .48) (Table 2). See Figure 1 to visualise DT’s performance in relation to matched controls.

**Table 1:** Test of Deficit results and BF values for accuracy (HSAM versus 20 matched controls).


TASK	HSAM	CONTROLS	*t*	ESTIMATED % OF CONTROL POPULATION ABOVE DT’S SCORE		ESTIMATED EFFECT SIZE (Z_CASE-CONTROLS_)		*p*	BF
		
	ACCURACY (%)	MEAN ACCURACY (%) (SD)		(%)	(95% CI)	POINT	(95% CI)		

*Simon Task*									

Compatible	100	97.97 (2.42)	0.82	21.16	8.94–37.53	0.84	0.32–1.34	0.19	3.11

Incompatible	97	92.10 (5.20)	0.92	18.47	7.14–34.33	0.94	0.40–1.47	0.19	2.97

*Part Set Cueing*									

Phase 1	58.33	50.41 (15.35)	0.50	31.02	16.38–48.33	0.52	0.04–0.98	0.25	4.50

Phase 2	62.50	50.00 (17.98)	0.68	25.28	11.94–42.21	0.70	0.20–1.18	0.25	4.04

Phase 3	75.00	48.75 (18.5)	1.39	9.11	2.08–21.72	1.42	0.78–2.04	0.09	4.23

Go/No-Go*									

Overall	99.50	95.50 (3.82)	1.02	16.00	5.61–31.27	1.05	0.49–1.59	0.16	2.56

*HSCT*									

Phase 1	100	99.65 (1.60)	0.21	41.66	25.41–59.04	0.22	–0.23–0.66	0.42	4.46

Phase 2	85.71	76.96 (12.66)	0.67	25.41	12.02–42.37	0.70	0.19–1.17	0.25	3.58

*Stroop Task*									

Phase 2	100	99.16 (0.55)	1.50	7.62	1.50–19.34	1.52	0.87–2.17	0.08	4.11

Phase 3	100	98 (0.75)	2.67	0.87	0.02–4.37	2.66	1.71–3.61	0.01^1^	3.30

*OSARI*									

Stop Trial	77.08	69.23 (6.80)	1.12	13.75	4.34–28.35	1.51	0.57–1.71	0.14	2.34


*Abbreviations*. Bayes Factor (BF), Hayling Sentence Completion (HSCT), Open-Source Anticipated Response Inhibition Task (OSARI). *Please note due to technical issues the Go/No-Go comparisons are between HSAM and 19 controls. ^1^Please note this value is not significant when accounting for multiple testing.

**Table 2:** Test of Deficit results and BF values for response times (RT) (HSAM vs. controls).


TASK	HSAM	CONTROLS	*T*	ESTIMATED % OF CONTROL POPULATION BELOW DT’S SCORE		ESTIMATED EFFECT SIZE (Z_CASE-CONTROLS_)		*p*	BF
		
	RT (S)	MEAN RT (S) (SD)		(%)	(95% CI)	POINT	(95% CI)		

*Simon Task*									

Compatible	0.48	0.45 (0.66)	0.04	51.75	34.64–68.57	0.05	–0.40–0.48	0.52	3.46

Incompatible	0.54	0.49 (0.68)	0.07	52.82	35.68–69.56	0.07	–0.37–0.51	0.53	2.27

*Go/No-Go*									

Overall	0.35	0.34 (0.02)	0	50.00	33.06–66.94	0	–0.44–0.44	0.50	4.47

*HSCT*									

Phase 1	1.02	1.48 (0.38)	–1.18	12.60	3.73–26.83	–1.21	–1.78––0.62	0.13	2.20

Phase 2	3.70	2.88 (1.04)	0.77	77.45	60.89–90.06	0.78	0.28–1.28	0.77	3.29

*Stroop Task*									

Phase 1	23.70	17.12 (4.18)	1.53	92.96	81.62–98.71	1.57	0.90–2.23	0.93	1.34

Phase 2	19.01	17.23 (3.00)	0.58	71.53	54.30–85.66	0.60	0.11–1.07	0.72	3.82

Phase 3	25.38	25.00 (4.21)	0.09	53.46	36.31–70.14	0.09	–0.35 –0.53	0.53	4.56

*OSARI*									

Go Trial	0.84	0.80 (0.02)	1.95	96.7	88.91–99.71	2.00	1.22–2.76	0.97	2.45

Stop Trial	0.76	0.78 (0.06)	0.76	77.45	60.89–90.06	0.78	0.28–1.28	0.77	3.96


*Abbreviations*. Bayes Factor (BF), Hayling Sentence Completion (HSCT), Open-Source Anticipated Response Inhibition Task (OSARI). *Please note due to technical issues the Go/No-Go comparisons are between HSAM and 19 controls.

OCI-R scores revealed both DT (score = 10) and the control sample (M = 16.44, SD = 8.29) were in the non-OCD range, and there was no significant difference between the groups in their scores, *t*(19) = –0.76, *p* = .46, BF = 3.35. Similarly, DT (score = 20) and the control sample (M = 17.60, SD = 5.87) scored in the non-autistic range, and the results of both groups were comparable, *t*(19) = 0.40, *p* = .70, BF = 3.96.

## Discussion

In the present study we explored for the first time the cognitive inhibition capabilities of a HSAM case with no clinical abnormalities. A battery of tasks assessing various domains of inhibition (e.g., memory inhibition, motor response suppression) was administered to DT and 20 matched controls, to test the hypothesis that augmented inhibitory processes contribute to HSAM. Countering our hypotheses, results showed that, overall, inhibitory processes in HSAM are comparable to those of normal individuals.

As described above, the self-memory system theory of autobiographical memory (Conway & Pleydell-Pearce, 2000) argues that whilst an array of different memories are constantly activated, few enter one’s consciousness, due to suppression of goal irrelevant information by executive control processes (Barzykowski et al., 2019). The amount DT remembers is clearly superior to the general population (Talbot et al., 2022), yet he reports to possess an authority over his own memory retrieval, which was predicted to be due to more efficient control systems. Crucially, whilst DT did not excel, we provide evidence that his cognitive inhibition is certainly not impaired. Many HSAM participants show signs of OCD (e.g., Parker et al., 2006), leading some researchers to theorise that the phenomenon is sustained by increased consolidation of autobiographical memories as a direct result of abnormal rehearsal (LePort et al., 2016; LePort et al., 2017). Studies in OCD populations have shown that this condition is associated with reduced cognitive inhibition, including a diminished ability to inhibit cognitive interference (Penadés et al., 2007). If OCD was a prerequisite of HSAM, then a performance deficit would likely have been observed in DT in this study. DT provides further evidence that HSAM can also exist in the absence of OCD (De Marco et al., 2021; Mazzoni et al., 2019; Santangelo et al., 2021) and indicates that the OCD hypothesis of HSAM may not be the best explanation of this ability, at least not in all described cases.

Moreover, our results, which are supported by Bayesian analysis, are consistent with evidence against the cognitive inhibition dependency hypothesis. Barzykowski et al. (2019) directly tested this theory, finding that depletion of inhibition resources did *not* affect the frequency of involuntary memories (and involuntary future thoughts). The authors discussed a contrasting explanation which may instead explain why humans are not flooded by mental time travel, that could also be relevant to the study of HSAM. Berntsen et al. (2013) have proposed the notion of *cue overload* as a mechanism modulating memory pop ups. This principle argues that for each cue there are multiple memories which are associated (Mazzoni et al., 2014; Vannucci et al., 2014; Uzer, 2016); when there are more events related to a specific cue, the cue is less likely to trigger the involuntary autobiographical memory, due to a stimuli overload. HSAM literature supports that these individuals remember significantly more unique events than most individuals. Various methodologies have been utilised to attempt to quantify exactly how many events are retained in HSAM. Most frequently, dates are presented as cues and participants are instructed to provide detailed memories, alongside specific information which can be verified (Frithsen et al., 2018; LePort et al., 2012; LePort et al., 2016; LePort et al., 2017). The number of dates administered is vast; BB, a young British HSAM male with no clinical diagnoses, successfully provided detailed memories for 88.4% of 500 dates administered (Mazzoni et al., 2019). Moreover, to ensure consistency, studies have presented the same dates again to participants, after substantial temporal delays, and participants have been found to reliably share the same events with 100% accuracy (Ally et al., 2013; Parker et al., 2006). In this line, if cognitive inhibition is not enhanced, perhaps then, it is the abundance of memories which keeps reliving at bay in HSAM, due to cue overload. Future HSAM research should consider exploring involuntary autobiographical memories, comparing both the frequency and characteristics of these spontaneous memory representations to test the cue overload hypothesis directly. A combination of laboratory (Barzykowski et al., 2022) and naturalistic studies (Schlagman & Kvavilashvili, 2008, for a recent discussion see Clevinger & Mace, 2024) could be extremely beneficial. Whilst literature has frequently measured voluntary retrieval in HSAM (e.g., LePort et al., 2016), no study has explored involuntary retrieval.

Our results support the growing body of literature showing HSAM enhancements are entirely specific to the functioning of autobiographical memory. In relation to intelligence, HSAM individuals are within the normal range (Parker et al., 2006; Patihis, 2015) and they appear to produce false memories as frequently (Patihis et al., 2013). Similarly, their creative thinking (Daviddi et al., 2022) and working memory are usual (LePort et al., 2012). Our findings indicate that this pattern extends to cognitive inhibition. At present, relatively little is known about HSAM due to the limited publications on this topic, with almost 50% of such publications employing single case designs (only 20 experimental articles according to a recent systematic review, Talbot, Convertino et al., 2024). Therefore, null results can still be considered informative, particularly as they were consistently found across a broad battery of inhibitory tasks. Dismissing lines of research which are less relevant, can help guide researchers towards lines of study which are most promising.

Whilst it seems cognitive inhibition is not a suitable explanation to explain control over inhibition in HSAM, it should also be acknowledged that our results could be (partially) impacted by ceiling effects, which could prevent reliable between group differences being revealed. Paradigms designed to assess general cognitive functioning are not always suitable for exceptional memory research, as HSAM individuals perform far beyond the task limits. To overcome this problem in future studies, various steps could be taken. Firstly, novel tasks could be devised which are specifically designed to have levels of difficulty which extend far beyond the “usual” limit. In this line, HSAM screening tasks have already been specifically designed which are too difficult for normal individuals to compete (LePort et al., 2012), and researchers are beginning to design novel tasks (Ford et al., 2022) that are successfully able to show the full spectrum of performance. Alternatively, existing tasks could be modified to increase difficulty and thus, error rate. For example, trial numbers could be increased, or dual tasks could be introduced, to cause greater cognitive load for participants.

The breadth of inhibition tasks used was deliberate (e.g., verbal, written, motor, memory), as it was important to strike a balance between maintaining full attention of participants (i.e., the testing session not being too long) and testing inhibition broadly. We predicted findings would be consistent across different inhibition measures if this explanation was theoretically robust. However, HSAM is an enhancement of *memory*, and it should be mentioned that this battery included very few memory-based inhibitory tasks. The task which most explicitly tested memory (part set cueing) was found to be far from ceiling for accuracy performance, indicating no meaningful performance differences, but it cannot be discounted that additional memory inhibitory tasks (e.g., retrieval-induced forgetting, Somos et al., 2022) would be beneficial in any future works.

Furthermore, it is necessary to state that this study is limited to the investigation of a single HSAM participant. Whilst this sample size is often a necessity in this area, due to the rareness of HSAM, single-case designs substantially reduce the generalisability of research findings. It cannot be ruled out that replicating this study in a larger HSAM cohort (e.g., a cohort the size of the work by Levine et al. (2021) could provide more informative findings. A larger sample replication could also provide stronger support that “normal” inhibition is a feature of HSAM as a whole. We emphasise that our study is limited to the conclusion that *DT’s* hyper memory is likely sustained by a process unrelated to cognitive inhibition. It is also important to stress here that cognitive inhibitory processes can be altered in the presence of psychopathology, and thus, potential future replications should control for within HSAM sample clinical differences (e.g., OCD symptom differences).

In conclusion, whilst it is undisputed that HSAM individuals excel at their memories, the present study indicates that this process is not supported by the help of enhanced cognitive inhibition.

## Data Accessibility Statement

As this work presents data from a single case that could be easily identifiable, the data and code has not been made publicly available. However, the data that support the findings of this study are available from the corresponding author, J.T., upon reasonable request.

## References

[1] Agam, Y., Joseph, R. M., Barton, J. J. S., & Manoach, D. S. (2010). Reduced cognitive control of response inhibition by the anterior cingulate cortex in autism spectrum disorders. NeuroImage, 52(1), 336–347. 10.1016/j.neuroimage.2010.04.010 20394829 PMC2883672

[2] Ally, B. A., Hussey, E. P., & Donahue, M. J. (2013). A case of hyperthymesia: Rethinking the role of the amygdala in autobiographical memory. Neurocase, 19(2), 166–181. 10.1080/13554794.2011.654225 22519463 PMC3432421

[3] Anderson, M. C., & Hanslmayr, S. (2014). Neural mechanisms of motivated forgetting. Trends in Cognitive Sciences, 18(6), 279–292. 10.1016/j.tics.2014.03.002 24747000 PMC4045208

[4] Anderson, M. C., & Hulbert, J. C. (2021). Active Forgetting: Adaptation of Memory by Prefrontal Control. Annual Review of Psychology, 72(1), 1–36. 10.1146/annurev-psych-072720-094140 32928060

[5] Andrés, P., & Howard, C. E. (2011). Part set cuing in older adults: Further evidence of intact forgetting in aging. Aging, Neuropsychology, and Cognition, 18(4), 385–395. 10.1080/13825585.2010.542892 21728887

[6] Baron-Cohen, S., Wheelwright, S., Skinner, R., Martin, J., & Clubley, E. (2001). Journal of Autism and Developmental Disorders, 31(1), 5–17. 10.1023/a:1005653411471 11439754

[7] Barzykowski, K., Hajdas, S., Radel, R., & Kvavilashvili, L. (2022). Effects of inhibitory control capacity and cognitive load on involuntary past and future thoughts: A laboratory study. Consciousness and Cognition, 102, 103353. 10.1016/j.concog.2022.103353 35642842

[8] Barzykowski, K., & Mazzoni, G. (2021). Do intuitive ideas of the qualities that should characterize involuntary and voluntary memories affect their classification? Psychological Research, 86(1), 170–195. 10.1007/s00426-020-01465-3 33582862 PMC8821514

[9] Barzykowski, K., Radel, R., Niedźwieńska, A., & Kvavilashvili, L. (2019). Why are we not flooded by involuntary thoughts about the past and future? testing the cognitive inhibition dependency hypothesis. Psychological Research, 83(4), 666–683. 10.1007/s00426-018-1120-6 30483873 PMC6529375

[10] Benjamin, D. J., Berger, J. O., Johannesson, M., Nosek, B. A., Wagenmakers, E. J., Berk, R., … & Johnson, V. E. (2018). Redefine statistical significance. Nature human behaviour, 2(1), 6–10. 10.1038/s41562-017-0189-z 30980045

[11] Berntsen, D. (2009). Spontaneous autobiographical memories. An introduction to the unbidden past. New York: Cambridge University Press. 10.1017/CBO9780511575921

[12] Berntsen, D. (2010). The unbidden past. Current Directions in Psychological Science, 19(3), 138–142. 10.1177/0963721410370301

[13] Berntsen, D., Staugaard, S. R., & Sørensen, L. M. T. (2013). Why am I remembering this now? Predicting the occurrence of involuntary (spontaneous) episodic memories. Journal of experimental psychology: General, 142(2), 426. 10.1037/a0029128 22746701

[14] Brown, N. R. (2016). Transition theory: A minimalist perspective on the organization of autobiographical memory. Journal of Applied Research in Memory and Cognition, 5(2), 128–134. 10.1016/j.jarmac.2016.03.005

[15] Brown, N. R. (2021). The possible effects of the COVID-19 pandemic on the contents and organization of autobiographical memory: A Transition-Theory perspective. Cognition, 212, 104694. 10.1016/j.cognition.2021.104694 33798951 PMC9748850

[16] Brown, N. R. (2023). Autobiographical memory and the self: A transition theory perspective. WIREs Cognitive Science, 14(3). 10.1002/wcs.1621 36189848

[17] Burgess, P. W., & Shallice, T. (1996). Response suppression, initiation and strategy use following frontal lobe lesions. Neuropsychologia, 34(4), 263–272. 10.1016/0028-3932(95)00104-2 8657357

[18] Clevinger, A. M., & Mace, J. H. (2024). Studying naturally occurring involuntary autobiographical memories with the diary approach: A comparison of diary methods. Applied Cognitive Psychology, 38(4). 10.1002/acp.4225

[19] Conway, M. A., & Pleydell-Pearce, C. W. (2000). The construction of autobiographical memories in the self-memory system. Psychological Review, 107(2), 261–288. 10.1037//0033-295x.107.2.261 10789197

[20] Crawford, J. R., & Garthwaite, P. H. (2012). Single-case research in Neuropsychology: A comparison of five forms of T-test for comparing a case to controls. Cortex, 48(8), 1009–1016. 10.1016/j.cortex.2011.06.021 21843884

[21] Crawford, J. R., Garthwaite, P. H., Azzalini, A., Howell, D. C., & Laws, K. R. (2006). Testing for a deficit in single-case studies: Effects of departures from normality. Neuropsychologia, 44(4), 666–677. 10.1016/j.neuropsychologia.2005.06.001 16046228

[22] Crawford, J. R., Garthwaite, P. H., & Porter, S. (2010). Point and interval estimates of effect sizes for the case-controls design in neuropsychology: Rationale, methods, implementations, and proposed reporting standards. Cognitive Neuropsychology, 27(3), 245–260. 10.1080/02643294.2010.513967 20936548

[23] Crawford, J. R., Garthwaite, P. H., & Ryan, K. (2011). Comparing a single case to a control sample: Testing for neuropsychological deficits and dissociations in the presence of covariates. Cortex, 47(10), 1166–1178. 10.1016/j.cortex.2011.02.017 21458788

[24] Crawford, J. R., & Howell, D. C. (1998). Comparing an individual’s test score against norms derived from small samples. The Clinical Neuropsychologist, 12(4), 482–486. 10.1076/clin.12.4.482.7241

[25] Daviddi, S., Orwig, W., Palmiero, M., Campolongo, P., Schacter, D. L., & Santangelo, V. (2022). Individuals with highly superior autobiographical memory do not show Enhanced creative thinking. Memory, 30(9), 1148–1157. 10.1080/09658211.2022.2094416 35786156

[26] De Marco, M., Iavarone, A., Santoro, G., & Carlomagno, S. (2015). Brief report: Two day-date processing methods in an autistic savant calendar calculator. Journal of Autism and Developmental Disorders, 46(3), 1096–1102. 10.1007/s10803-015-2626-z 26476739

[27] De Marco, M., Mazzoni, G., Manca, R., & Venneri, A. (2021). Functional neural architecture supporting highly superior autobiographical memory. Brain Connectivity. 10.1089/brain.2020.0858 33403914

[28] Dienes, Z. (2014). Using Bayes to get the most out of non-significant results. Frontiers in psychology, 5, 781. 10.3389/fpsyg.2014.00781 25120503 PMC4114196

[29] Donders, F. C. (1969). Over de snelheid van psychische processen [On the speed of mental processes] (W. Koster, Trans.). In W. G. Koster (Ed.), Attention and performance II (pp. 412–431). Amsterdam: North Holland. (Original work published 1868). 10.1016/0001-6918(69)90065-1

[30] Dresler, M., Shirer, W. R., Konrad, B. N., Müller, N. C. J., Wagner, I. C., Fernández, G., Czisch, M., & Greicius, M. D. (2017). Mnemonic training reshapes brain networks to support superior memory. Neuron, 93(5). 10.1016/j.neuron.2017.02.003 PMC5439266 28279356

[31] Enright, S. J., & Beech, A. R. (1993). Reduced cognitive inhibition in obsessive—compulsive disorder. British Journal of Clinical Psychology, 32(1), 67–74. 10.1111/j.2044-8260.1993.tb01028.x 8467275

[32] Fawcett, J. M., & Hulbert, J. C. (2020). The many faces of forgetting: Toward a constructive view of forgetting in everyday life. Journal of Applied Research in Memory and Cognition, 9(1), 1–18. 10.1016/j.jarmac.2019.11.002

[33] Finnbogadóttir, H., & Berntsen, D. (2013). Involuntary future projections are as frequent as involuntary memories, but more positive. Consciousness and Cognition, 22(1), 272–280. 10.1016/j.concog.2012.06.014 22884775

[34] Foa, E. B., Huppert, J. D., Leiberg, S., Langner, R., Kichic, R., Hajcak, G., & Salkovskis, P. M. (2002). The obsessive-compulsive inventory: Development and validation of a short version. Psychological Assessment, 14(4), 485–496. 10.1037/1040-3590.14.4.485 12501574

[35] Ford, L., Shaw, T. B., Mattingley, J. B., & Robinson, G. A. (2022). Enhanced semantic memory in a case of highly superior autobiographical memory. Cortex, 151, 1–14. 10.1016/j.cortex.2022.02.007 35378418

[36] Frithsen, A., Stark, S. M., & Stark, C. E. (2018). Response bias, recollection, and familiarity in individuals with highly superior autobiographical memory (HSAM). Memory, 27(6), 739–749. 10.1080/09658211.2018.1561896 30596537

[37] Gibson, E. C., Ford, L., & Robinson, G. A. (2022). Investigating the role of future thinking in a case of highly superior autobiographical memory. Cortex, 149, 188–201. 10.1016/j.cortex.2022.01.016 35272062

[38] Gohier, B., Ferracci, L., Surguladze, S. A., Lawrence, E., El Hage, W., Kefi, M. Z., Allain, P., Garre, J.-B., & Le Gall, D. (2009). Cognitive inhibition and working memory in Unipolar Depression. Journal of Affective Disorders, 116(1–2), 100–105. 10.1016/j.jad.2008.10.028 19042027

[39] Hasher, L., Lustig, C., & Zacks, R. T. (2007). Inhibitory mechanisms and the control of attention. In A. Conway, C. Jarrold, M. Kane & J. Towse (Eds.), Variation in working memory (pp. 227–249). New York: Oxford University Press. 10.1093/acprof:oso/9780195168648.003.0009

[40] Hasher, L., & Zacks, R. T. (1988). Working memory, comprehension, and aging: A review and a new view. The Psychology of Learning and Motivation, 22,193–225. 10.1016/S0079-7421(08)60041-9

[41] He, J., Hirst, R., Puri, R., Coxon, J. P., Byblow, W. D., Hinder, M. R., Skippen, P., Matzke, D., Heathcote, A., Wadsley, C., Silk, T., Hyde, C., Parmar, D., Pedapati, E., Gilbert, D., Huddleston, D., Mostofsky, S. H., Leunissen, I., MacDonald, H., … Puts, N. (2022). Osari, an Open-Source Anticipated Response Inhibition Task. 10.31234/osf.io/fzdp2 PMC9170665 34751923

[42] Huber, S., Klein, E., Moeller, K., & Willmes, K. (2015). Comparing a single case to a control group–applying linear mixed effects models to repeated measures data. Cortex, 71, 148–159. 10.1016/j.cortex.2015.06.020 26218619

[43] Hughes, J. E. A., Ward, J., Gruffydd, E., Baron-Cohen, S., Smith, P., Allison, C., & Simner, J. (2018). Savant syndrome has a distinct psychological profile in autism. Molecular Autism, 9(1), 53. 10.1186/s13229-018-0237-1 30344992 PMC6186137

[44] Laughland, A., & Kvavilashvili, L. (2024). The frequency and cueing mechanisms of involuntary autobiographical memories while driving. Memory, 1–15. 10.1080/09658211.2023.2296826 38166488

[45] LePort, A. K. R., Mattfeld, A. T., Dickinson-Anson, H., Fallon, J. H., Stark, C. E. L., Kruggel, F., Cahill, L., & McGaugh, J. L. (2012). Behavioral and neuroanatomical investigation of highly superior autobiographical memory (HSAM). Neurobiology of Learning and Memory, 98(1), 78–92. 10.1016/j.nlm.2012.05.002 22652113 PMC3764458

[46] LePort, A. K. R., Stark, S. M., McGaugh, J. L., & Stark, C. E. L. (2017). A cognitive assessment of highly superior autobiographical memory. Memory, 25(2), 276–288. 10.1080/09658211.2016.1160126 26982996 PMC5488704

[47] LePort, A. K., Stark, S. M., McGaugh, J. L., & Stark, C. E. (2016). Highly superior autobiographical memory: Quality and quantity of retention over time. Frontiers in Psychology, 6. 10.3389/fpsyg.2015.02017 PMC4720782 26834661

[48] Levine, L. J., Murphy, G., Lench, H. C., Greene, C. M., Loftus, E. F., Tinti, C., Schmidt, S., Muzzulini, B., Grady, R. H., Stark, S. M., & Stark, C. E. L. (2021). Remembering facts versus feelings in the wake of political events. Cognition and Emotion, 35(5), 936–955. 10.1080/02699931.2021.1910496 33829942

[49] Mace, J. H. (2007). Involuntary memory: Concept and theory. Involuntary Memory, 1–19. 10.1002/9780470774069.ch1

[50] Maxcey, A. M., Dezso, B., Megla, E., & Schneider, A. (2019). Unintentional forgetting is beyond cognitive control. Cognitive Research: Principles and Implications, 4(1). 10.1186/s41235-019-0180-5 PMC6635537 31313055

[51] Mazzoni, G., Clark, A., De Bartolo, A., Guerrini, C., Nahouli, Z., Duzzi, D., De Marco, M., McGeown, W., & Venneri, A. (2019). Brain activation in highly superior autobiographical memory: The role of the precuneus in the autobiographical memory retrieval network. Cortex, 120, 588–602. 10.1016/j.cortex.2019.02.020 30926140

[52] Mazzoni, G., Vannucci, M., & Batool, I. (2014). Manipulating cues in involuntary autobiographical memory: Verbal cues are more effective than pictorial cues. Memory & Cognition, 42(7), 1076–1085. 10.3758/s13421-014-0420-3 24871426

[53] Neath, A. A., & Cavanaugh, J. E. (2012). The Bayesian information criterion: background, derivation, and applications. Wiley Interdisciplinary Reviews: Computational Statistics, 4(2), 199–203. 10.1002/wics.199

[54] Neumann, N., Dubischar-Krivec, A. M., Braun, C., Löw, A., Poustka, F., Bölte, S., & Birbaumer, N. (2010). The mind of the mnemonists: An MEG and neuropsychological study of autistic memory savants. Behavioural Brain Research, 215(1), 114–121. 10.1016/j.bbr.2010.07.008 20637245

[55] Nørby, S. (2015). Why Forget? On the Adaptive Value of Memory Loss. Perspectives on Psychological Science, 10(5), 551–578. 10.1177/1745691615596787 26385996

[56] Palombo, D. J., Sheldon, S., & Levine, B. (2018). Individual differences in autobiographical memory. Trends in Cognitive Sciences, 22(7), 583–597. 10.1016/j.tics.2018.04.007 29807853

[57] Parker, E. S., Cahill, L., & McGaugh, J. L. (2006). A case of unusual autobiographical remembering. Neurocase, 12(1), 35–49. 10.1080/13554790500473680 16517514

[58] Patihis, L. (2015). Individual differences and correlates of highly superior autobiographical memory. Memory, 24(7), 961–978. 10.1080/09658211.2015.1061011 26314991

[59] Patihis, L., Frenda, S. J., LePort, A. K., Petersen, N., Nichols, R. M., Stark, C. E., McGaugh, J. L., & Loftus, E. F. (2013). False memories in highly superior autobiographical memory individuals. Proceedings of the National Academy of Sciences, 110(52), 20947–20952. 10.1073/pnas.1314373110 PMC3876244 24248358

[60] Peirce, J., Gray, J. R., Simpson, S., MacAskill, M., Höchenberger, R., Sogo, H., Kastman, E., & Lindeløv, J. K. (2019). Psychopy2: Experiments in behavior made easy. Behavior Research Methods, 51(1), 195–203. 10.3758/s13428-018-01193-y 30734206 PMC6420413

[61] Penadés, R., Catalán, R., Rubia, K., Andrés, S., Salamero, M., & Gastó, C. (2007). Impaired response inhibition in obsessive compulsive disorder. European Psychiatry, 22(6), 404–410. 10.1016/j.eurpsy.2006.05.001 17127038

[62] Rittmo, J. O., & McIntosh, R. D. (2021). singcar: Comparing single cases to small samples in R. Journal of Open Source Software, 6(68), 3887. 10.21105/joss.03887

[63] Robledo-Castro, C., Hederich-Martínez, C., & Castillo-Ossa, L. F. (2023). Cognitive stimulation of executive functions through computational thinking. Journal of Experimental Child Psychology, 235, 105738. 10.1016/j.jecp.2023.105738 37421925

[64] R Studio Team (2020). RStudio: Integrated Development for R. RStudio, PBC, Boston, MA. URL http://www.rstudio.com/

[65] Ruta, L., Mazzone, D., Mazzone, L., Wheelwright, S., & Baron-Cohen, S. (2012). The Autism-Spectrum Quotient—Italian Version: A Cross-Cultural Confirmation of the Broader Autism Phenotype. Journal of Autism and Developmental Disorders, 42(4), 625–633. 10.1007/s10803-011-1290-1 21626054

[66] Ruzich, E., Allison, C., Smith, P., Watson, P., Auyeung, B., Ring, H., & Baron-Cohen, S. (2015). Measuring autistic traits in the general population: a systematic review of the Autism-Spectrum Quotient (AQ) in a nonclinical population sample of 6,900 typical adult males and females. Molecular Autism, 6(1), 2. 10.1186/2040-2392-6-2 25874074 PMC4396128

[67] Santangelo, V., Cavallina, C., Colucci, P., Santori, A., Macrì, S., McGaugh, J. L., & Campolongo, P. (2018). Enhanced brain activity associated with memory access in highly superior autobiographical memory. Proceedings of the National Academy of Sciences, 115(30), 7795–7800. 10.1073/pnas.1802730115 PMC6064994 29987025

[68] Santangelo, V., Pedale, T., Colucci, P., Giulietti, G., Macrì, S., & Campolongo, P. (2021). Highly superior autobiographical memory in aging: A single case study. Cortex, 143, 267–280. 10.1016/j.cortex.2021.05.011 34167804

[69] Scarpazza, C., Costa, C., Battaglia, U., Berryessa, C., Bianchetti, M. L., Caggiu, I., Devinsky, O., Ferracuti, S., Focquaert, F., Forgione, A., Gilbert, F., Pennati, A., Pietrini, P., Rainero, I., Sartori, G., Swerdlow, R., & Camperio Ciani, A. S. (2023). Acquired pedophilia: International Delphi-Method-based consensus guidelines. Translational Psychiatry, 13(1). 10.1038/s41398-023-02314-8 PMC9849353 36653356

[70] Schacter, D. L. (2022). The seven sins of memory: an update. Memory, 30(1), 37–42. 10.1080/09658211.2021.1873391 33459149 PMC8285452

[71] Schlagman, S., & Kvavilashvili, L. (2008). Involuntary autobiographical memories in and outside the laboratory: How different are they from voluntary autobiographical memories? Memory & Cognition, 36(5), 920–932. 10.3758/MC.36.5.920 18630199

[72] Sica, C., Ghisi, M., Altoè, G., Chiri, L. R., Franceschini, S., Coradeschi, D., & Melli, G. (2009). The Italian version of the Obsessive Compulsive Inventory: Its psychometric properties on community and clinical samples. Journal of Anxiety Disorders, 23(2), 204–211. 10.1016/j.janxdis.2008.07.001 18701254

[73] Simon, J. R., & Rudell, A. P. (1967). Auditory SR compatibility: the effect of an irrelevant cue on information processing. Journal of applied psychology, 51(3), 300. 10.1037/h0020586 6045637

[74] Somos, E., Mazzoni, G., Gatti, D., & Jellema, T. (2022). “Be careful what you recall”: Retrieval-induced forgetting of genuine real-life autobiographical memories. Quarterly Journal of Experimental Psychology, 76(1), 84–92. 10.1177/17470218221078499 35073798

[75] Stroop, J. R. (1935). Studies of interference in serial verbal reactions. Journal of experimental psychology, 18(6), 643. 10.1037/h0054651

[76] Talbot, J., Convertino, G., De Marco, M., Venneri, A., & Mazzoni, G. (2024). Highly superior autobiographical memory (HSAM): A systematic review. Neuropsychology Review. 10.1007/s11065-024-09632-8 PMC11965258 38393540

[77] Talbot, J., Gatti, D., Boccalari, M., Marchetti, M., Mitaritonna, D., Convertino, G., Stockner, M., & Mazzoni, G. (2024). Dimensions of a hyper memory: investigating the factors modulating exceptional retrieval in a single case of highly superior autobiographical memory (HSAM). Memory, 1–11. 10.1080/09658211.2024.2351576 38727555

[78] Talbot, J., Gatti, D., Mitaritonna, D., Marchetti, M., Convertino, G., & Mazzoni, G. (2022). Stimulating a hyper memory: A single case TMS study on an individual with highly superior autobiographical memory. Brain Stimulation, 15(5), 1122–1124. 10.1016/j.brs.2022.08.006 35985471

[79] Uzer, T. (2016). Retrieving autobiographical memories: How different retrieval strategies associated with different cues explain reaction time differences. Acta Psychologica, 164, 144–150. 10.1016/j.actpsy.2016.01.004 26802518

[80] Vannucci, M., Batool, I., Pelagatti, C., & Mazzoni, G. (2014). Modifying the frequency and characteristics of involuntary autobiographical memories. PLoS ONE, 9(4). 10.1371/journal.pone.0089582 PMC3981656 24717536

[81] Vannucci, M., Pelagatti, C., Hanczakowski, M., Mazzoni, G., & Paccani, C. R. (2015). Why are we not flooded by involuntary autobiographical memories? few cues are more effective than many. Psychological Research, 79(6), 1077–1085. 10.1007/s00426-014-0632-y 25468208

[82] Wagenmakers, E. J. (2007). A practical solution to the pervasive problems of *p* values. Psychonomic Bulletin & Review, 14, 779–804 10.3758/BF03194105 18087943

[83] Wheelwright, S., Auyeung, B., Allison, C., & Baron-Cohen, S. (2010). Defining the broader, medium and narrow autism phenotype among parents using the Autism Spectrum Quotient (AQ). Molecular Autism, 1(1), 10. 10.1186/2040-2392-1-10 20678260 PMC2913943

